# Supplementary material for: Ferroelectric Smectic C Liquid Crystal Phase with Spontaneous Polarization in the Direction of the Director
Source: Adv Sci (Weinh). 2024 Oct 22;11(45):2409827. doi: 10.1002/advs.202409827 (PMC11615755; doi:10.1002/advs.202409827)
Supplement: Supplementary file 1 — Supporting Information [file ADVS-11-2409827-s001.docx]

Supporting Information

Ferroelectric Smectic C Liquid Crystal Phase with Spontaneous Polarization in the Direction of the Director

Hirotsugu Kikuchi*, Hiroya Nishikawa, Hiroyuki Matsukizono, Shunpei Iino, Takeharu Sugiyama, Toshio Ishioka, Yasushi Okumura*


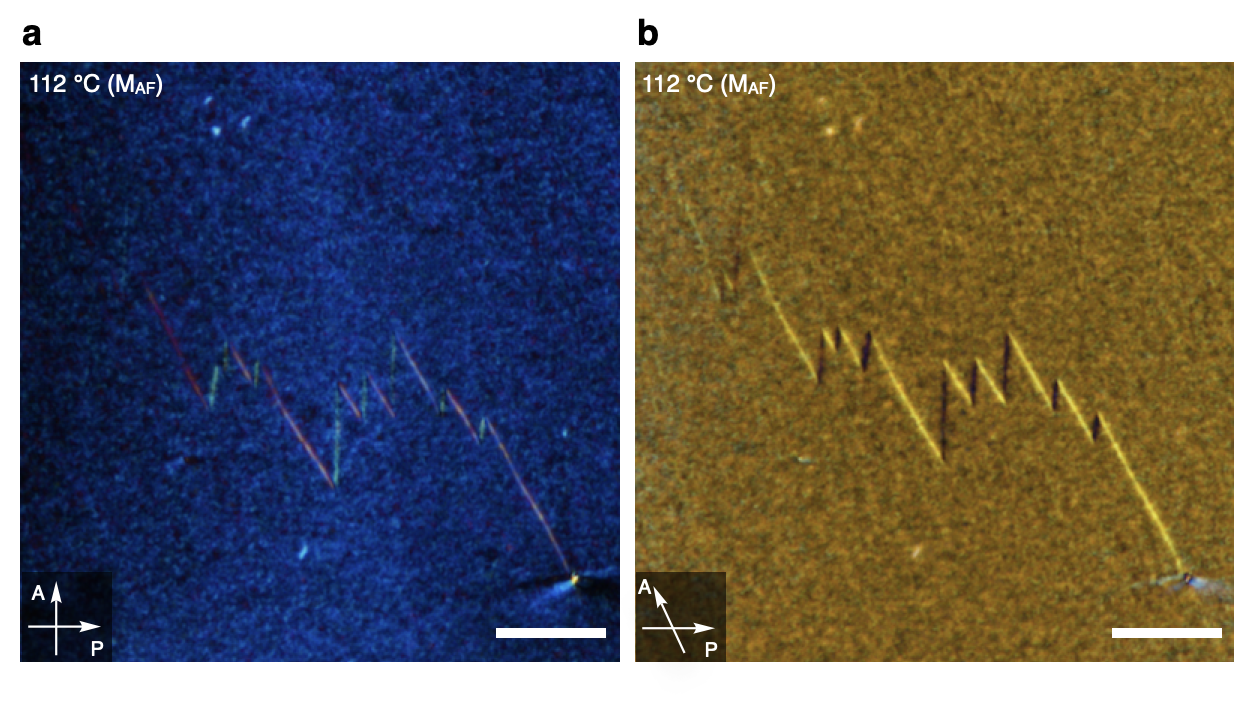


**Figure S1**. POM images for the M_AF_ phase (112 °C) in a polyimide cell (4 μm) under crossed- (a) and decrossed-(b) polarizers. Scale bar: 200 μm.

**
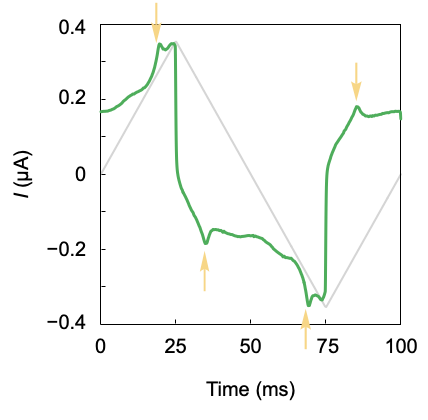
**

**Figure S2**. Polarization reversal current measurement in the M_AF_ phase (112 °C) under the *E*-field with a triangular voltage wave (400 V, 10 Hz). The orange arrows denote the polarization reversal current peaks.

**
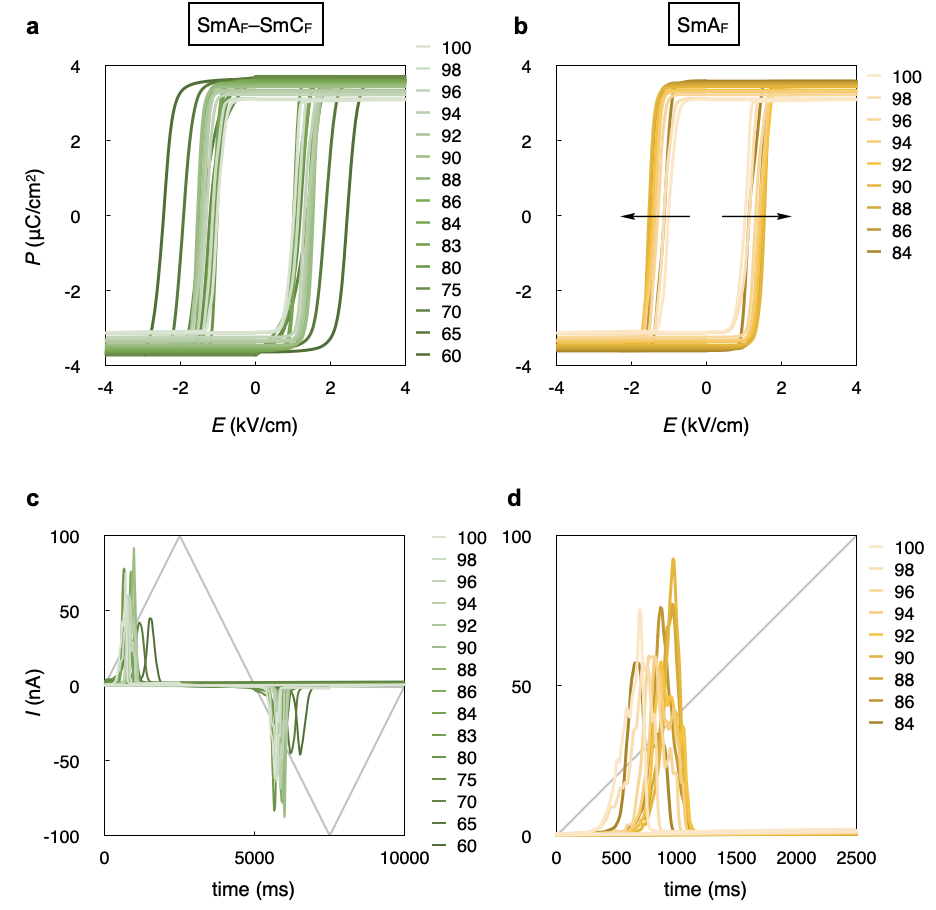
**

**Figure S3**. *D*–*E* hysteresis loops (a,b) and polarization reversal current profiles (c,d) for compound **1**. a,c ) SmA_F_ and SmC_F_ regimes, b,d) SmA_F_ regime. All data were recorded under the *E*-field with a triangular voltage wave (400 V, 0.1 Hz).

**
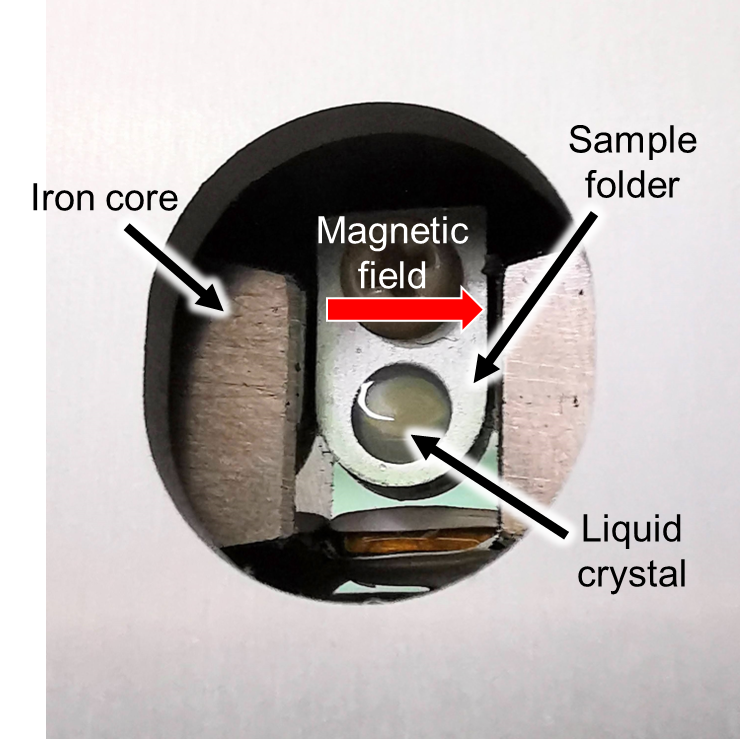
**

**Figure S4**. (a) Sample folder for liquid crystals mounted on the temperature stage box with magnetic circuit.

**
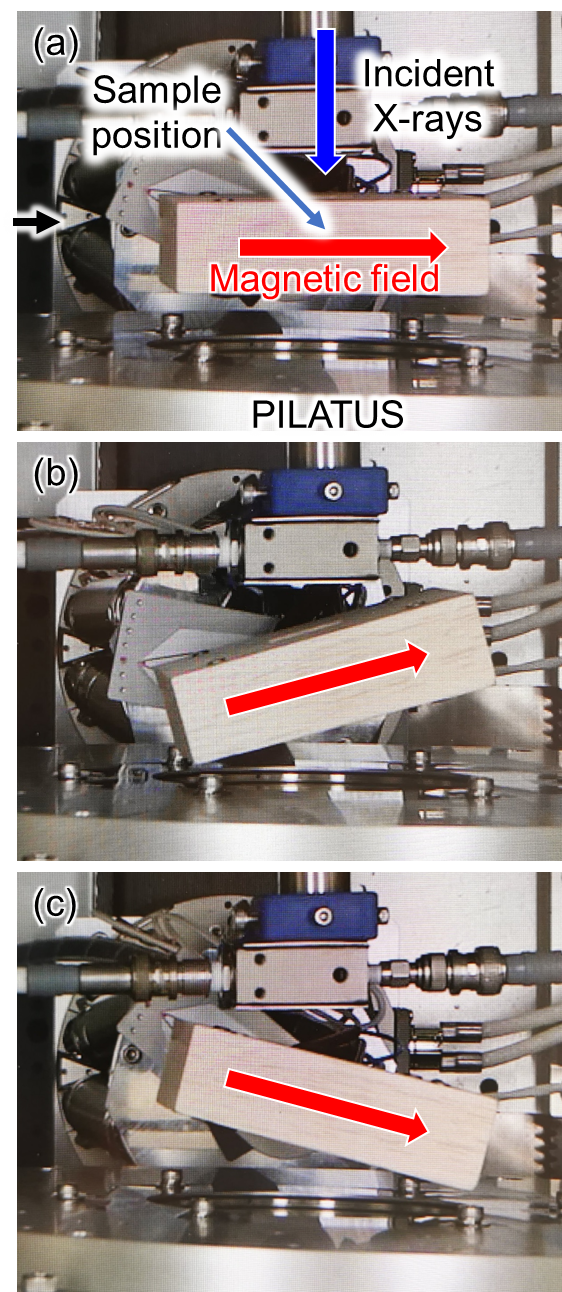
**

**Figure S5**. Rotation of the *M*-field application temperature stage box attached to the hexapods. Rotation angle = 0° (a), −15° (b), 15° (c).
